# Wnt/beta-catenin signaling activates microRNA-181 expression in hepatocellular carcinoma

**DOI:** 10.1186/2045-3701-1-4

**Published:** 2011-01-18

**Authors:** Junfang Ji, Taro Yamashita, Xin W Wang

**Affiliations:** 1 Liver Carcinogenesis Section, Laboratory of Human Carcinogenesis, Center for Cancer Research, National Cancer Institute, Bethesda, Maryland, USA; 2 Liver Disease Center and Kanazawa University Hospital, Kanazawa University, Kanazawa, Japan

## Abstract

**Background:**

Hepatocellular carcinoma (HCC) is a malignant cancer with an observable heterogeneity and microRNAs are functionally associated with the tumorigenesis of HCC. We recently identified that EpCAM (CD326)-positive cells isolated from alpha-fetoprotein (AFP)-positive HCC samples are hepatic cancer stem cells (HepCSCs). EpCAM^+^AFP^+ ^HepCSCs have an activated Wnt/β-catenin signaling with a parallel increased expression of all four microRNA-181 family members. We hypothesized that Wnt/β-catenin signaling transcriptionally activates microRNA-181s in HCC.

**Results:**

Using both western blot and quantitative reverse transcriptase-PCR analyses, we found that the expression of all four microRNA-181 family members was positively correlated with β-catenin expression in HCC cell lines. MicroRNA-181 expression could be directly induced upon an activation of Wnt/β-catenin signaling, which includes Wnt10B overexpression, inhibition of GSK3β signaling by LiCl, or forced expression of β-catenin/Tcf4. Moreover, microRNA-181 expression was inhibited upon an inactivation of Wnt/β-catenin signaling by an induction of adenomatosis polyposis coli (APC) expression or silencing β-catenin via RNA interference. In addition, seven putative β-catenin/Tcf4 binding sites were identified in the promoter region of the microRNA-181a-2 and microRNA-181b-2 transcripts. Consistently, we found that Tcf4 interacted with these regions *in vivo *using chromatin immunoprecipitation assay.

**Conclusions:**

Taken together, our results demonstrate that microRNA-181s are transcriptionally activated by the Wnt/beta-catenin signaling pathway in HCC.

## Background

Hepatocellular carcinoma (HCC) is the fifth most common malignant cancer and the third leading cause of cancer death worldwide with the observable heterogeneity in its morphology, clinical behaviour, and molecular profiles [1]. Studies on HCC molecular profiling have revealed many HCC-associated deregulated genes and signaling pathways, in which Wnt/β-catenin signaling has been proposed to be critical [2-9]. We recently found that isolated HCC cells using a cell surface marker EpCAM (CD326) from alpha-fetoprotein (AFP) positive HCC cases are hepatic cancer stem cells (HepCSCs), where an activation of Wnt/β-catenin signaling is a major feature [5].

MicroRNAs (miRNAs, miRs) are a class of small, non-coding, endogenous and functional RNAs. Mature miRNAs are generated from sequential processing of primary miRNA transcripts by Drosha and Dicer, then serve as posttranscriptional regulators of gene expression in certain biological events including carcinogenesis [10]. Interestingly, many miRNAs exist as a multi-member family indicating their functional redundancy. We recently found that the miR-181 family contains four highly conserved mature miR-181s, i.e., miR-181a, miR-181b, miR-181c and miR-181d, which are derived independently from 6 precursors located on 3 different chromosomes. They are miR-181a-1 and miR-181b-1 located on chromosome 1, miR-181a-2 and miR-181b-2 on chromosome 9, and miR-181c and miR-181d on chromosome 19. We also found that all of these microRNAs are highly expressed in EpCAM^+^AFP^+ ^HepCSCs [9,10]. In addition, we demonstrate that this family is critical in maintaining stemness of EpCAM^+^AFP^+ ^HepCSCs, in part by directly targeting an inhibitor of Wnt/β-catenin signaling (nemo-like kinase [NLK]) and two hepatic transcriptional regulators of differentiation, i.e., caudal type homeobox transcription factor 2 (CDX2) and GATA binding protein 6 (GATA6). We also found that inhibition of miR-181s leads to a reduction in quantity and tumor initiating ability of EpCAM^+^AFP^+ ^HepCSCs, suggesting the potential utility of these miRNAs as molecular targets of HepCSC [9,10]. The fact that all four independently encoded miR-181 transcripts are similarly activated to maintain "stemness" is intriguing, which implies that a common cellular signaling pathway may converge to activate miR-181s. In this study, we identified that Wnt/β-catenin signaling appears to be a common activator to induce miR-181s.

## Results

### Association of miR-181 expression with Wnt/β-catenin signaling activation

**Figure 1:**
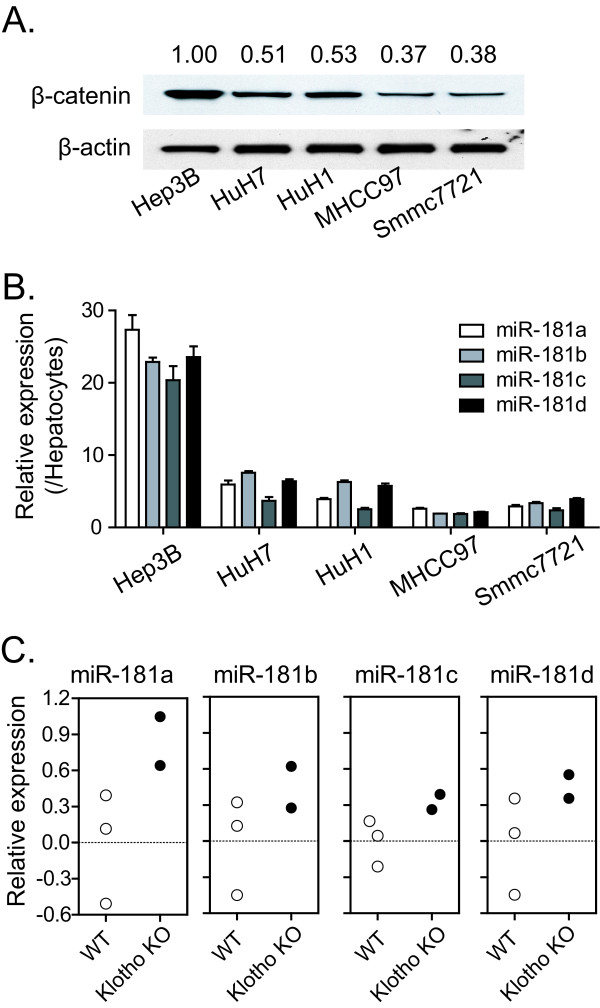
**Expression of miR-181 is associated with β-catenin level**. (A) Western blot analysis of β-catenin and β-actin in Hep3B, HuH7, HuH1, MHCC97 and Smmc7721 HCC cell lines. The relative intensity of β-catenin was listed on the top of individual western blot band. (B) qRT-PCR analysis of the four mature miR-181s in five HCC cell lines. Levels of miR-181s in these HCC cell lines were measured in triplicate and normalized by U6 level. After further normalization by the reading in adult primary human hepatocytes, the relative level for each miRNA was shown as the mean ± standard deviation. (C) qRT-PCR analysis of the four mature miR-181s in liver tissues from three wild-type mice and two Klotho knockout mice. miR-181s' level in these tissues was measured in triplicate, normalized to levels of U6.

To test whether miR-181s were transcriptional targets of Wnt/β-catenin, we first examined the expression of β-catenin and miR-181s in five different HCC cell lines. Figure 1A and 1B showed that β-catenin and miR-181s were concordantly expressed in these cell lines. Moreover, we tested the association of miR-181 and β-catenin using *Klotho *mice where loss of Klotho, a secreted Wnt antagonist, leads to β-catenin activation [11]. Consistently, levels of all 4 mature miR-181s in the liver tissues of *Klotho *mice were higher than those of wild-type mice (figure 1C). Therefore, both *in vivo *and *in vitro *data demonstrate that the expression of miR-181s is positively correlated with the Wnt/β-catenin signaling pathway.

### Wnt/β-catenin signaling activation induces the expression of all 4 mature miR-181s

**Figure 2:**
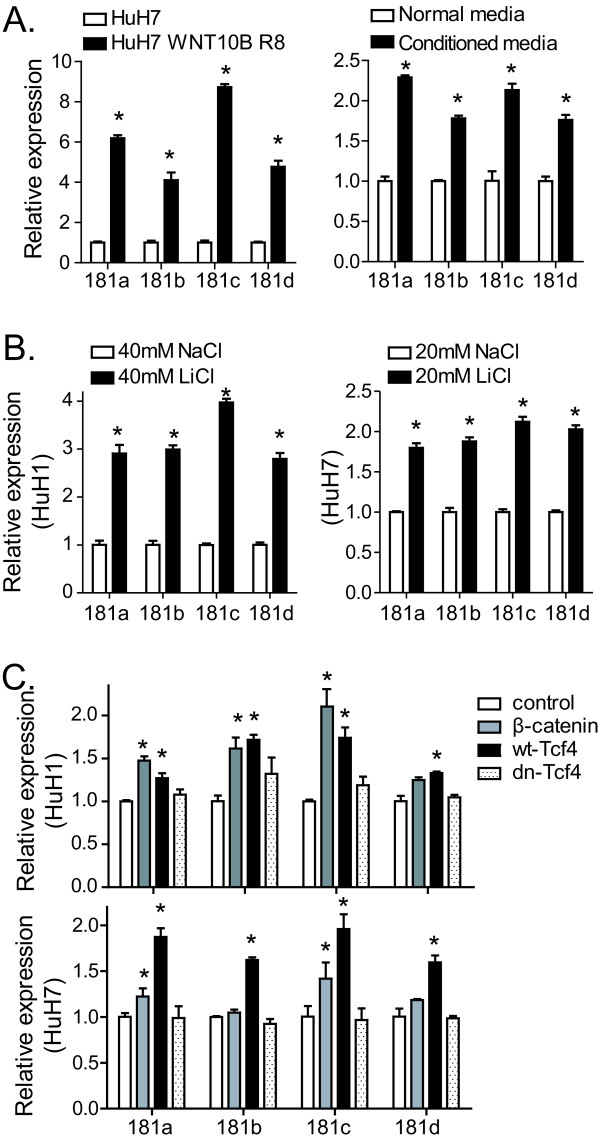
**Wnt/β-catenin signaling activation induces the level of miR-181**. (A) qRT-PCR analysis of all four mature miR-181s in HuH7 and HuH7 WNT10B R8 cells (left panel) and in HuH7 cells cultured with normal media or conditioned media from HuH7 WNT10B R8 for 3 days (right panel). (B) qRT-PCR of miR-181s in HuH1 and HuH7 cells incubated with LiCl or NaCl for 3 days. (C) qRT-PCR analysis of all four mature miR-181s in HuH7 cells and HuH1 cells with transient transfection of β-catenin, wild-type Tcf4 (wt-Tcf4), dominant negative Tcf4 (dn-Tcf4) or control for three days. The experiments were performed in triplicates and results were shown as mean ± standard deviation. * refers to p < 0.05.

To examine whether Wnt/β-catenin signaling could directly induce miR-181 expression, we first used HuH7 cells with stable WNT10B expression (HuH7 WNT10B R8) [12]. Compared to control HuH7 cells, HuH7 WNT10B R8 had 6.2-, 4.1-, 8.5-, and 4.4- fold higher levels of miR-181a, miR-181b, miR-181c and miR-181d, respectively (figure 2A). Moreover, conditioned media derived from HuH7 WNT10B R8 also significantly induced the expression of miR-181s in HuH7 cells (figure 2A). Second, lithium chloride (LiCl), an activator of the Wnt/β-catenin pathway through inhibiting glycogen synthase kinase (GSK)-3β then consequently stabilizing β-catenin [13], was applied to further examine the role of Wnt/β-catenin pathway in regulating miR-181s. We found that LiCl significantly induced expression of all 4 miR-181 transcripts in HuH7 (figure 2B) and HuH1 cells (data not shown) when compared to NaCl control treated cells. Additionally, miR-181s were significantly induced by ectopic expression of a constitutively activated β-catenin or a wild type Tcf4, a co-transcriptional activator of β-catenin, in HuH7 (figure 2C) and HuH1 cells (data not shown). As a control, a dominant negative Tcf-4 mutant failed to do so. These data indicate that Wnt/β-catenin signaling activation directly induces miR-181 expression.

### Wnt/β-catenin signaling inhibition reduces the levels of miR-181s

**Figure 3:**
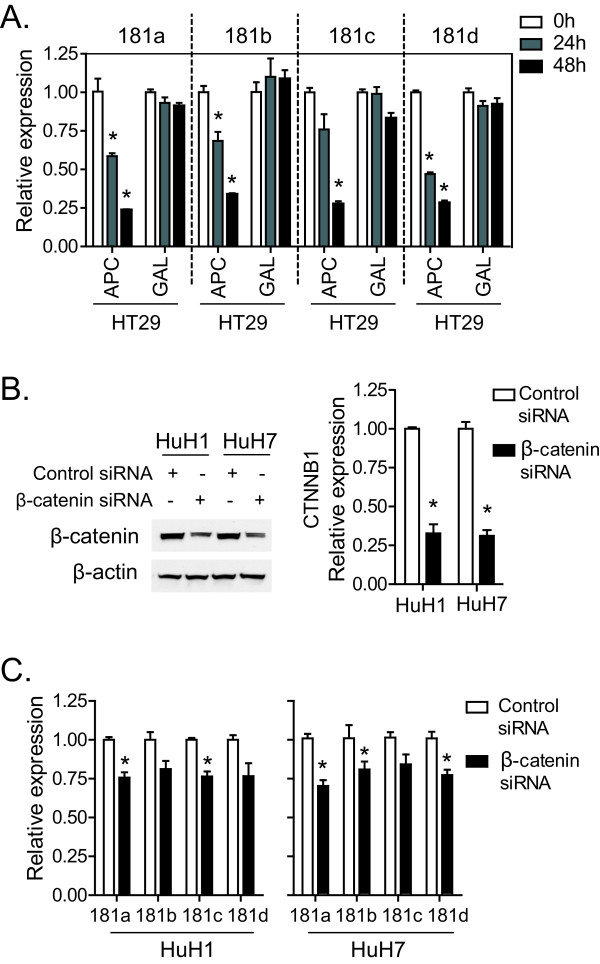
**Wnt/β-catenin signaling inhibition reduces the expression of miR-181**. (A) HT29-APC and HT29-GAL cells were incubated with 100 μM of ZnCL_2 _for different time points (0 hours, 24 hours and 48 hours). Then qRT-PCR analysis of all four mature miR-181s was performed in these cells. (B, C) β-catenin siRNA and control siRNA was transfected in HuH7 and HuH1 cells using lipofectamine 2000. Western blotting analysis of β-catenin and β-actin (left panel) and qRT-PCR analysis of CTNNB1 (right panel) were performed (B). qRT-PCR analysis of all four mature miR-181s (C). All these experiments were done in triplicates and the level of miRNAs and genes were shown as mean ± standard deviation. * refers to p < 0.05.

Several known proteins such as adenomatosis polyposis coli (APC) can promote β-catenin degradation [14]. To investigate the effect of Wnt/β-catenin inactivation on the expression of miR-181s, we used HT29-APC cells where the APC gene is under the control of a zinc-activated metallothionein promoter [14]. The levels of all four miR-181s were significantly decreased in HT29-APC cells following addition of zinc chloride (ZnCl_2_), but not in control HT29-GAL cells (figure 3A). Furthermore, β-catenin siRNA was employed to suppress Wnt/β-catenin activity in HuH1 and HuH7 cells. Consistently, declined β-catenin expression by siRNA (Figure 3B and 3C) significantly reduced the expression of all four miR-181 transcripts (figure 3D). These data demonstrate that an inactivation of Wnt/β-catenin signaling results in an inhibition of miR-181 expression.

### Tcf4 binds to the promoter of miR-181a-2/miR-181b-2 transcript

**Figure 4:**
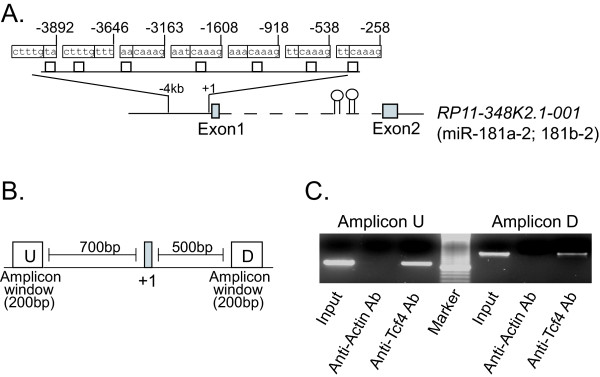
**Tcf4 associates with the promoter of miR-181s**. (A) Predicted Tcf4 binding sites in the promoter region of the miR-181a-2/miR-181b-2 gene. (B) PCR amplicons for ChIP assay were designed within 200-bp windows in the ~700-bp upstream of the transcription start site (amplicon U), and in the ~500-bp downstream of transcription start site (amplicon D). (C) PCR analysis of Tcf4 chromatin immunoprecipitate. Actin chromatin immunoprecipitate was used as negative control. PCR products were run on a 3.0% agarose gel.

Studies have shown that the β-catenin/Tcf complex transcriptionally regulates their target gene expression through binding to a consensus core TCF/LEF-binding site (5'-A/T A/T CAAAG-3') within the promoter regions [15,16]. GenBank database analyses indicate that miR-181a-1 and miR-181b-1 are located in the intron 2 of a novel host gene (RP11-31E23.1-001) on 1q31 (data not shown), while miR-181a-2 and miR-181b-2 are located in the intron 2 of a novel transcript, RP11-348K2.1-001, on 9q33 (figure 4A). We searched the core TCF/LEF binding site within 4kb genomic sequences of the predicted promoter regions of these two genes. Seven TCF/LEF binding elements were identified in the promoter region of RP11-348K2.1-001 (figure 4A), and three elements were in the promoter region of RP11-31E23.1-001 (data not shown). MiR-181c and miR-181d are present in the intergenic region of chromosome 19 and consequently no promoter region is available for analysis. Furthermore, RP11-348K2.1-001 was selected for chromatin immuoprecipitation (ChIP) assay using Tcf4 antibody in Hep3B cells where both β-catenin and miR-181s are highly elevated. We designed two amplicons within a 200-bp window, i.e., amplicon U (in the upstream of transcription site) and amplicon D (in the downstream of transcription site) (figure 4B). The results showed that Tcf4 specifically bound to both amplicons in the miR-181a-2/miR-181b-2 promoter (figure 4C), indicating that the miR-181a-2/miR-181b-2 transcript is a direct transcriptional target of Wnt/β-catenin canonical signaling pathway in HCC.

## Discussion

HCC is an aggressive cancer with extensive clinical and genomic heterogeneity. We have previously utilized a combination of EpCAM and AFP expression to identify two distinct HCC subgroups, i.e., EpCAM^+^AFP^+ ^HCCs (named as HpSC-HCCs) with poor prognosis, high metastasis, and the presence of a hepatic stem cell (HpSC) feature, and EpCAM^-^AFP^- ^HCCs (named as MH-HCCs) with good prognosis and the presence of a mature hepatocytes (MH) feature. We have experimentally demonstrated that EpCAM^+^AFP^+ ^HCC cells are HepCSCs [3,5,15]. Interestingly, cDNA and microRNA array analyses indicate that Wnt/β-catenin signaling is activated and all four miR-181 family members are highly expressed in both HpSC-HCC clinical specimens and in isolated HepCSCs [5,9]. These results suggest that Wnt/β-catenin might be a key regulator of miR-181. The exploration of the molecular link between Wnt/β-catenin and miR-181 would further increase our understanding of the intricate molecular regulation in cancer stem cells and facilitate the designing of effective strategies to eliminate cancer stem cells, which are thought to be responsible for tumour relapse and drug resistance.

Our current study has demonstrated that the Wnt/β-catenin signaling pathway can directly regulate miR-181s expression in HCC. This conclusion is based on the following observations: 1) miR-181 expression was positively associated with β-catenin level in both HCC cell lines and mouse livers where β-catenin is augmented; 2) activation of Wnt/β-catenin signaling directly induced miR-181 expression, while inhibition of Wnt pathway directly reduced miR-181 expression; 3) we found evidence of *in vivo *binding between the Tcf4/β-catenin complex and the TCF/LEF binding elements in the promoter region of miR-181a-2/miR-181b-2 transcript.

Wnt/β-catenin is a key molecular signaling regulator in embryonic stem cells and in a variety of human cancers including HCC. Similarly, Wnt/β-catenin signaling is known to be involved in liver development and regeneration, and has been shown to be activated in EpCAM^+ ^HepCSCs [5,17-19]. Interestingly, miR-181s maintain the stemness of HepCSCs partially through suppressing NLK, an inhibitor of Wnt/β-catenin signaling, to consequently induce the transcriptional activity of Tcf4/β-catenin [10]. Our findings revealed a positive feedback regulatory loop between miR-181 and Wnt/β-catenin signaling in maintaining stemness of HepCSCs where either miR-181s or Wnt/β-catenin can promote stemness by targeting the pool of their downstream genes. A future challenge will be to explore this positive feedback loop as a molecular target to eradicate hepatic cancer stem cells.

## Conclusions

The Wnt/beta-catenin signaling is a major transcriptional regulator of miR-181s expression.

## Methods

### Plasmids, small interfering RNAs (siRNAs), antibodies and chemicals

Constitutively active β-catenin expression vector (pCI-NEO-βcateninXL) (mutated at S33Y), wild-type Tcf4 expression vector (pcDNA/myc-hTcf4) and dominant negative Tcf4 expression vector (pcDNA/myc-ΔN-hTcf4) were generous gifts from Dr. Bert Vogelstein [20]. β-catenin siRNA and negative control siRNA were purchased from Qiagen. Anti-β-catenin monoclonal antibody (BD Transduction Laboratories), anti-β-actin monoclonal antibody (Sigma) and anti-Tcf-4 monoclonal antibody (Chemicon) were used in this study. Lithium chloride, sodium chloride and zinc chloride were commercially available (Sigma).

### Cells, conditioned medium, transfection and mice liver tissues

Human HCC cell lines (Hep3B, HuH7, HuH1, MHCC97 and Smmc7721) were cultured as previously described [6]. HT29-APC, HT29-GAL cells were kindly provided by Dr. Bert Vogelstein and routinely cultured as described [14,15]. To induce APC's expression, we incubated HT29-APC cells with 100 μmol/L of zinc chloride for 24 and 48 hours. HuH7 cells with stably expressed WNT10B (WNT10B clone R8) were maintained in our lab [12]. WNT10B conditioned media were prepared from HuH7 WNT10B R8. Three wild-type and two *Klotho *knock out mice were kindly given by Dr. Toren Finkel. Liver tissues were dissected from these mice and immediately stored at -80°C until RNA extraction. Transfection of siRNAs and plasmids was done using Lipofectamine 2000 (Invitrogen) according to the manufacturer's instrument. A total of 100nM siRNAs or 2 μg/ml plasmids were used for each transfection. Total RNA was extracted three days after transfection.

### RNA extraction and quantitative real-time reverse transcription PCR (qRT-PCR)

Total RNA was extracted using Trizol (Invitrogen). Reverse transcription was done using High Capacity cDNA Reverse Transcription Kit for mRNAs and TaqMan MicroRNA Reverse Transcription Kit for miRNAs. The expression of mRNAs and mature miRNAs was measured using Taqman Gene Assay specific for CTNNB1 and Taqman MicroRNA Assays specific for miR-181a, miR-181b, miR-181c and miR-181d (Applied biosystems). The experiments were performed in triplicate. The Taqman gene assay for 18S and Taqman MicroRNA Assay for U6 RNA were used to normalize the relative abundance of mRNA and miRNA, respectively.

### Protein extraction and western blotting

Total protein was extracted as previously described [6]. They were electrophoresed on 10% Tris-Glycine Gel (Invitrogen) and transferred to nitrocellulose membrane (Invitrogen). The membrane was incubated with anti-β-catenin and anti-β-actin, followed by anti-mouse IgG conjugated to horseradish peroxidase. The bands were developed using enhanced chemiluminescence detection reagents (Amersham).

### Chromatin immuoprecipitation (ChIP)

ChIP assay was done using the Chromatin Immunoprecipitation Assay Kit (Upstate), Hep3B cells and anti-Tcf4 antibody according to the manufactures' instrument. The oligonucleotides flanking transcription start site and predicted Tcf4 binding sites were designed within ~200-bp windows in the 700-bp upstream of the transcription start site (amplicon U), and in the 500-bp windows downstream of transcription start site (amplicon D) for detecting miR-181a-2 and miR-181b-2 promoter. The primers were 5'-aaa caa agc aac tgc cat gta ct-3' (sense) and 5'-agg gtc ctt ggg gtt tat ttt-3' (antisense) for amplicon U, and 5'-ggt tgc ttt cca ttc ttt gc-3' (sense) and 5'-ttt ggc cac aca aaa cta gag at-3' (antisense) for amplicon D. The PCR was done using Platinum Taq Polymerase High Fidelity (Invitrogen).

### Statistical analyses

Student's t-test (two-tailed) was used for statistical analysis of comparative data between different groups. p < 0.05 was considered to be statistically significant. Data were presented as the mean ± standard deviation (S.D.) and the analyses were performed using GraphPad Prism software 5.0 (GraphPad Software, San Diego, CA).

## Competing interests

The authors declare that they have no competing interests.

## Authors' contributions

JJ contributed to experiment design, performing experiments, data analysis and manuscript preparation. TY contributed to the data analysis and XWW contributed to experimental design, data analysis and manuscript preparation. All authors have read and approved the final manuscript.
